# Bone mineral density in high-level endurance runners: Part B—genotype-dependent characteristics

**DOI:** 10.1007/s00421-021-04789-z

**Published:** 2021-09-22

**Authors:** A. J. Herbert, A. G. Williams, S. J. Lockey, R. M. Erskine, C. Sale, P. J. Hennis, S. H. Day, G. K. Stebbings

**Affiliations:** 1 School of Health Sciences, Birmingham City University, Birmingham, UK; 2 Sports Genomics Laboratory, Department of Sport and Exercise Sciences, Manchester Metropolitan University, Manchester, UK; 3 Faculty of Health, Education, Medicine and Social Care, Anglia Ruskin University, Chelmsford, UK; 4 School of Sport and Exercise Science, Liverpool John Moores University, Liverpool, UK; 5 Musculoskeletal Physiology Research Group, Sport, Health and Performance Enhancement Research Centre, School of Science and Technology, Nottingham Trent University, Nottingham, UK; 6 School of Medicine and Clinical Practice, University of Wolverhampton, Wolverhampton, UK; 7 Institute of Sport, Exercise and Health, University College London, London, UK

**Keywords:** Genetics, Single-nucleotide polymorphisms, Bone mineral density, Endurance, Marathon, Mechanical loading

## Abstract

**Purpose:**

Inter-individual variability in bone mineral density (BMD) exists within and between endurance runners and non-athletes, probably in part due to differing genetic profiles. Certainty is lacking, however, regarding which genetic variants may contribute to BMD in endurance runners and if specific genotypes are sensitive to environmental factors, such as mechanical loading via training.

**Method:**

Ten single-nucleotide polymorphisms (SNPs) were identified from previous genome-wide and/or candidate gene association studies that have a functional effect on bone physiology. The aims of this study were to investigate (1) associations between genotype at those 10 SNPs and bone phenotypes in high-level endurance runners, and (2) interactions between genotype and athlete status on bone phenotypes.

**Results:**

Female runners with *P2RX7* rs3751143 AA genotype had 4% higher total-body BMD and 5% higher leg BMD than AC + CC genotypes. Male runners with *WNT16* rs3801387 AA genotype had 14% lower lumbar spine BMD than AA genotype non-athletes, whilst AG + GG genotype runners also had 5% higher leg BMD than AG + GG genotype non-athletes.

**Conclusion:**

We report novel associations between *P2RX7* rs3751143 genotype and BMD in female runners, whilst differences in BMD between male runners and non-athletes with the same *WNT16* rs3801387 genotype existed, highlighting a potential genetic interaction with factors common in endurance runners, such as high levels of mechanical loading. These findings contribute to our knowledge of the genetic associations with BMD and improve our understanding of why some runners have lower BMD than others.

**Supplementary Information:**

The online version contains supplementary material available at 10.1007/s00421-021-04789-z.

## Introduction

Individuals who complete higher levels of weight-bearing physical activity tend to have higher bone mineral density (BMD) (Warburton et al. 2006). Despite this, some athletes, such as endurance runners may be at risk of low BMD and increased risk of stress fracture injury, which negatively impacts both health and performance (Pollock et al. 2010). Excessive training volumes and/or dietary restriction undertaken by this population can result in reduced energy availability, which can negatively impact bone metabolism and potentially reduce BMD (Papageorgiou et al. 2018).

Nonetheless, inter-individual variability in bone phenotypes exist, even within sport-specific cohorts (as demonstrated in Part A), which may be explained in part by genetic factors. Heritability of BMD is reportedly 50–80% (Ralston and Uitterlinden 2010), with 98 loci having been associated with total, femoral neck and lumbar spine dual-energy X-ray absorptiometry (DXA)-derived BMD previously (Trajanoska et al. 2019). Few investigations, have replicated these associations independently or considered gene-environment interactions (Trajanoska et al. 2019). It remains unclear whether certain genes may be sensitive to mechanical loading from physical activity and what the outcome is of such an interaction for BMD and injury risk (Herbert et al. 2019).

Mitchell et al. (2016) investigated genomic components of bone phenotypes and the relationship with physical activity using SNPs that had previously been associated with BMD via GWAS (Estrada et al. 2012). The authors observed nominal interactions at the lumbar spine in children between physical activity and variants such as Wnt family member 16 (*WNT16*) rs3801387 and axin 1 (*AXIN1*) rs9921222. In athletes competing in weight-bearing sports specifically, higher total-body BMD was shown in the vitamin D receptor (*VDR*) FokI rs2228570 GG and GA, but not AA, genotypes compared to non-athlete controls (Nakamura et al. 2002b). Interestingly, within swimmers, a lower total BMD was observed in the GG genotype when compared to non-athlete controls (Nakamura et al. 2002b). Together, these findings suggest that individuals with the GG genotype may be more responsive to mechanical loading, resulting in greater BMD under conditions of high mechanical loading, but lower BMD when the mechanical loading is less, such as in swimming. Thus, phenotypes can vary between individuals of the same genotype, especially when that genotype demonstrates sensitivity to mechanical loading.

It is possible that athletes tend to possess advantageous variants of genes that are responsive to mechanical loading and others that are important for the attainment of peak BMD. This may result in a competitive advantage via more consistent and higher volume training because of the higher BMD and subsequent reduced injury risk. Some endurance runners are at risk of reduced energy availability due to the potential undertaking of high training volumes and/or insufficient energy intake, which may negatively impact BMD. Consequently, possessing an advantageous genetic predisposition for BMD is likely to be of greater importance for the health and performance of populations at risk of low BMD, such as endurance runners, than athletic populations competing in sports that exhibit high peak and multi-directional forces on bone. Those who possess a more advantageous genotype may have a greater response to loading, resulting in higher BMD and a reduced risk of a stress fracture. The potential consequence of a disadvantageous genotype has been demonstrated in dancers, who are also at risk of low BMD. Specifically, genetic variants in the oestrogen receptor and the Wnt/β-catenin pathways were associated with an increased prevalence of low BMD in elite dancers (Amorim et al. 2018). Moreover, the increased prevalence of low BMD in the dancers was not predicted by previously suggested risk factors of body mass, menstrual disturbances and energy availability. Thus, low BMD in the absence of known risk factors further emphasises the potential modulation of the BMD phenotype via genetic characteristics (Amorim et al. 2018).

Only a small number of BMD-associated genetic variants have been explored in athletic populations, or in relation to gene-physical activity interactions, so the genetic influence on BMD in athletic populations is still unknown. Endurance runners, in particular, experience high volumes of mechanical loading at certain sites (e.g. tibia) but less loading at others (e.g. lumbar spine). Despite experiencing high volumes of loading, some endurance runners may have low BMD and thus present a suitable population in which to investigate gene-physical activity interactions vis-à-vis BMD (Herbert et al. 2019). Variability as well as differences in bone phenotypes between high-level endurance runners and non-athletes are reported in Part A of this two-part investigation. The purpose of this second part was to (1) investigate whether the 10 SNPs, individually and collectively, associated with phenotypes including total-body BMD (_T_BMD), leg BMD (_L_BMD), lumbar spine BMD (_LS_BMD), total-body T-score and total-body Z-score in high-level endurance runners; (2) investigate whether being an endurance runner or non-athlete affected any association between genotype and bone phenotypes. The 10 SNPs chosen for investigation all have reported functional effects on bone and have also been identified via genome-wide association study and/or candidate gene association study as being associated with BMD (Table 1). We hypothesised associations between genotype and bone phenotypes in high-level endurance runners, plus the existence of genotype-athlete status interactions on bone phenotypes due to the impact of long-term mechanical loading in runners.

**Table 1:** Gene functions and previous associations of SNP with bone phenotypes and/or metabolism

Variant	Gene function	SNP association
*axin 1* (*AXIN1*) rs9921222	Encodes regulators of the WNT signalling pathway, specifically as an element of the beta-catenin destruction complex (Baron and Kneissel 2013), and therefore, may influence bone mass by stimulating differentiation and replication of osteoblasts to enhance bone formation	T-allele associated with lower femoral neck and lumbar spine BMD via meta-analysis (Estrada et al. 2012). The rs9921222 SNP is also suggested to interact with physical activity to nominally associate with BMD (Mitchell et al. 2016)
*BDNF antisense RNA* (*BDNF-AS*) rs6265	*BDNF* knockdown has been shown to inhibit osteoblast differentiation, resulting in increased bone formation (Guo et al. 2016)	TT genotype is associated with lower spine and hip BMD in Caucasians (Deng et al. 2013)
Collagen type I alpha 1 chain (*COL1A1*) rs1800012	Encodes Collagen type 1, which is the most abundant protein in bone. Functional studies have shown that the rs1800012 polymorphism is associated with alterations in COL1A1 transcription and protein production, influencing bone mass (Mann and Ralston 2003)	AA (TT) homozygotes associated with lower hip and lumbar spine BMD as well as increased risk of fracture in meta-analysis (Jin et al. 2011). Contradictory findings, reporting no association, however, have been observed (Garcia‐Giralt et al. 2002)
Catechol-O-methyltransferase (*COMT*) rs4680	Catalyses the methylation of catechol oestrogens to methoxy oestrogens (inactive metabolites) and thus, lower COMT enzyme activity (AA genotype) results in higher levels of 16-hydroxy-oestradiol, which retains oestrogenic activity and enhances BMD oestrogens and consequently influence BMD (Eriksson et al. 2005; Lorentzon et al. 2007)	AA genotype associated with higher BMD than GG genotypes in girls aged 10–12 years (Eriksson et al. 2005) but prevalence of fracture has been reported to be higher in AA genotype elderly men in comparison with the GG genotype (Eriksson et al. 2008). The SNP also modulates the association between physical activity and BMD (Lorentzon et al. 2007)
LDL receptor related protein 5 (*LRP5*) rs3736228	Activation of Wnt/β-catenin signaling, via Wnt binding to LRP5 proteins increases the sensitivity of osteoblasts to mechanical loading (Krishnan et al. 2006; Robinson et al. 2006)	The T-allele has been associated with lower BMD and increased fracture risk in GWAS (Estrada et al. 2012) and the TT genotype also influences the effects of physical activity on spine BMD in men (Kiel et al. 2007)
Purinergic receptor P2X 7 (*P2RX7*) rs3751143	Expressed in osteoblasts, osteoclasts and osteocytes. In vitro studies have shown P2RX7 activation both inhibits bone resorption through osteoclast apoptosis and increases mineralisation through osteoblast differentiation (Wesselius et al. 2013)	Lower hip and lumbar spine BMD associated with the CC loss of function genotype in women (Wesselius et al. 2013). The C allele has also been associated with stress fracture incidence in military recruits and elite athletes (Varley et al. 2016)
TNF receptor superfamily member 11a (*TNFRSF11A*) rs3018362	Encodes RANK which portrays a pivotal role in osteoclast differentiation and function (Albagha et al. 2010)	A-allele associated with lower BMD via GWAS (Albagha et al. 2010) and cortical bone in adolescents (Paternoster et al. 2010). The SNP is also suggested to influence stress fracture incidence in elite athletes (Varley et al. 2015)
TNF receptor superfamily member 11b (*TNFRSF11B*) rs4355801	Encodes OPG (osteoprotegerin) which is secreted by osteoblasts and aids in regulating osteoclast differentiation, thus negatively regulating resorption (Simonet et al. 1997)	A-allele associated with lower BMD via GWAS (Richards et al. 2008) and cortical BMD in adolescents (Paternoster et al. 2010)
Vitamin D receptor (*VDR*) rs2228570	*VDR* controls the transcription of other genes including osteocalcin, which are instrumental for calcium absorption and bone formation (Moran et al. 2014)	Higher total body BMD observed in GG genotypes in weight-bearing athletes compared to controls. Potential influence on BMD via a mechanical loading genotype interaction also observed (Nakamura et al. 2002a, b)
Wnt family member 16 (*WNT16*) rs3801387	Wnt16 can signal via both canonical and non-canonical pathways and is a key regulator of osteoblast-to-osteoclast communication and subsequently influence bone mass (Gori et al. 2015)	The A-allele has been associated with lower lumbar spine and femoral neck BMD and osteoporotic fracture via GWAS (Estrada et al. 2012) and/or candidate gene association studies (Hendrickx et al. 2014). *WNT* rs3801387 has also been reported to interact with physical activity to demonstrate nominal associations with BMD (Mitchell et al. 2016)

## Materials and methods

### Participants and participant recruitment

Participants comprised 103 high-level endurance runners (45 men, 58 women) and 112 (52 men, 60 women) ethnically matched non-athletes. Briefly, runners were included if they had completed at least one official long-distance event (≥ 3000 m) faster than a pre-determined threshold time (Table 1, Part A) and were considered national/international standard. Please see Part A for a full description of participant characteristics. All experimental procedures were conducted in accordance with the guidelines in the Declaration of Helsinki and approved by the local Ethics Committee of Manchester Metropolitan University.

### Protocol

All runners completed a questionnaire that detailed geographic ancestry, performance, training practices, injury and sporting history, whilst female runners also provided menstruation history. Non-athletes completed a questionnaire in relation to ethnicity, general health and physical activity level to establish matched ethnic ancestry and ensure no history of high-level sporting competition. DXA scans on all participants were completed following the manufacturer’s guidelines to obtain _T_BMD, _L_BMD, _LS_BMD, total-body T- and Z-scores.

All participants also provided either a blood, saliva or buccal swab sample, from which DNA was extracted and genotyped for the 10 SNPs, which were selected according to the volume and strength of evidence in the literature of their association with BMD and/or biological function/mechanism in relation to BMD (Table 1).

For blood, a 5 mL sample was collected from a superficial forearm vein into EDTA collection tubes and stored in 1.5 mL microcentrifuge tubes (Eppendorf AG, Hamburg, Germany) at − 20 °C. Saliva samples were collected following a minimum 30-min abstinence from food and drink into Oragene DNA OG-500 collection tubes (DNA Genotek Inc., Ontario, Canada) in accordance with the manufacturer’s guidelines before being stored at room temperature. For buccal cell sample collection, participants brushed one OmniSwab collection tip (Whatman Sterile OmniSwab, GE Healthcare, USA) against the inside of one cheek for 30 s before repeating this with a second swab on the opposite cheek to obtain two samples (following the same abstinence as saliva samples) before being stored at − 20 °C in a 2 mL microcentrifuge tube. DNA was extracted using the Qiagen QIAcube spin protocol (Qiagen, Crawley, UK) and the Qiagen DNA Blood Mini Kit (Qiagen) for whole blood, saliva and buccal samples in accordance with the manufacturer’s guidelines. Approximately 75% of participant DNA was obtained from blood, 23% from saliva and 2% via buccal swabs.

All participants were genotyped for the 10 SNPs using the fluorophore-based detection technique of TaqMan real-time polymerase chain reaction (PCR) on either the Fluidigm EP1 (Fluidigm, Cambridge, UK) or StepOnePlus (Applied Biosystems, Paisley, UK). End-point fluorescence measurement of VIC and FAM determined the different genotypes for the 10 SNPs using the software supplied by the respective manufacturers of each PCR machine.

The majority of samples (95%) were genotyped via the Fluidigm EP1 by combining 2 μL GTXpress Master Mix (X2) (Applied Biosystems), 0.2 μL 20X Fast GT Sample Loading Reagent (Fluidigm), 0.2 μL nuclease-free H_2_O and 1.6 μL of purified DNA into each well of a 192 × 24 microchip. Negative controls were placed into 4 wells on each 192 × 24 microchip, in which nuclease-free H_2_O replaced the DNA sample. 1.78 μL assay (20X) (Applied Biosystems), 1.78 μL 2X Assay Loading Reagent (Fluidigm) and 0.18 μL ROX reference dye (Invitrogen, Paisley, UK) were combined per assay inlet. An integrated fluid circuit controller RX (Fluidigm) was used to mix samples and assays using a Load Mix (166X) script. PCR was performed using a real-time FC1 Cycler (Fluidigm) GT 192 × 24 Fast v1 protocol. Denaturation began at 95 °C for 120 s followed by 45 cycles of incubation at 95 °C for 2 s and then annealing and extension at 60 °C for 20 s before end-point analysis was completed in the EP1 reader. Genotyping was performed with the Fluidigm SNP genotyping analysis software.

The remaining 5% of samples were genotyped by combining 5 μL Genotyping Master Mix or GTXpress Master Mix (Applied Biosystems), 4.3 μL H_2_O, 0.5 μL assay (Applied Biosystems), and 0.2 μL of purified DNA (~ 9 ng), for samples derived from blood and saliva into wells on a 96-well plate (MicroAmp EnduraPlate Optical 96-Well Clear Reaction Plate, Applied Biosystems). For DNA taken from buccal swabs, 5 μL Genotyping Master Mix was combined with 3.5 μL H_2_O, 0.5 μL assay mix, and 1 μL DNA solution (~ 9 ng DNA). Negative controls were also placed into 2 wells on each 96-well plate, in which nuclease-free H_2_O replaced the DNA sample. Each well on a 96-well plate contained a total reaction volume of ~ 10 μL before being covered with an optical seal (MicroAmp Optical Adhesive Film, Applied Biosystems). PCR was performed using a StepOnePlus Real-Time PCR system (Applied Biosystems). For GTXpress Master Mix, an initial 20 s at 95 °C was followed by 50 cycles of denaturation for 3 s at 95 °C, then annealing and extension at 60 °C for 20 s. For Genotyping Master Mix, denaturation began at 95 °C for 10 min, with 40 cycles of incubation at 92 °C for 15 s and then annealing and extension at 60 °C for 1 min. Genotyping analysis was performed with StepOnePlus software version 2.3.

All samples were analysed in duplicate and were in 100% agreement. Similarly, there was 100% agreement between the StepOnePlus and Fluidigm PCR systems as determined by the analysis of one variant on 94 samples.

### Statistical analysis

Using additive (AA vs. Aa vs. aa), dominant (AA + Aa vs. aa) and recessive (AA vs. Aa + aa) genetic models for men and women separately, analysis of variance (ANOVA) was implemented to investigate associations between the 10 SNPs individually, and collectively as a total genotype score (TGS), and bone phenotypes (_T_BMD, _L_BMD, _LS_BMD, T-score, Z-score) in runners. Recessive and dominant models were executed with respect to the allele considered disadvantageous for BMD. For TGS, each SNP was allocated scores according to existing literature (Williams and Folland 2008), where the homozygote associated with higher BMD was given a score of 2, the heterozygotes scored 1 and the other homozygote given 0. The total score was then transformed to lie within 0–100 (e.g. TGS = 100/20 × (2 + 1 + 0 + 1 + 1 + 1 + 0 + 2 + 1 + 2) = 55). Consequently, participants were allocated to either a “low” (≤ 55), “moderate” (60–70) or “high” (≥ 75) TGS group based upon calculated TGS score. Significant associations from any additive model were further explored using Bonferroni post-hoc tests. ANOVA was also used to explore interactions between the 10 SNPs, individually and collectively, and athlete status on bone phenotypes. Following any significant interaction between athlete status and genotype for a bone phenotype, simple main effects with pairwise comparisons were conducted to analyse cohort-dependent differences in these bone phenotypes between runners and non-athletes across the same genotype. To control for Type 1 statistical errors from multiple testing, a false discovery rate (FDR) of 0.2 (Benjamini and Hochberg 1995) was applied within each family of ANOVAs for each SNP and uncorrected *P*-values are reported except where stated. Consequently, two FDR models for each SNP (each including 15 *P*-values for every ANOVA implemented) were applied to both the analyses within the runners and the analyses exploring the interactions between genotype and athlete status on bone phenotype. Due to an insufficient number of participants with two copies of the minor allele, *COL1A1* rs1800012 female analyses only included 5 *P*-values for each FDR model whilst *LRP5* rs3736228 male analyses only included 10 *P*-values for one FDR model (interaction analyses). Alpha was set at 0.05 and data are reported as mean (SD).

## Results

Participant characteristics and phenotype data for men and women are as described in Part A of this investigation.

### Genetic associations in runners

**Fig. 1:**
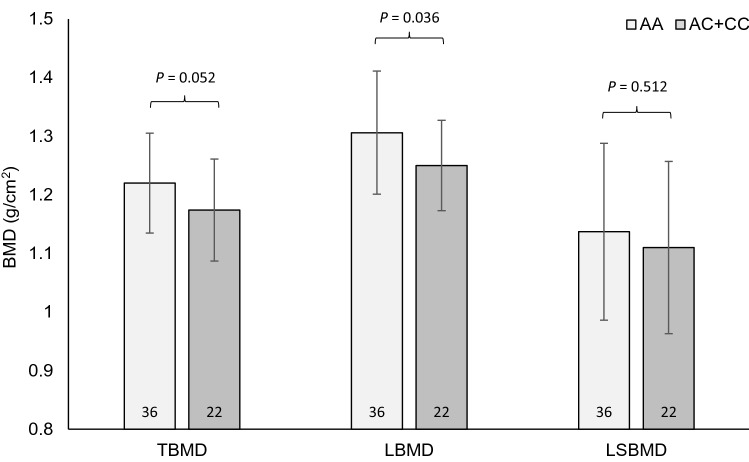
Mean total bone mineral density (_T_BMD), leg bone mineral density (_L_BMD) and lumbar spine bone mineral density (_LS_BMD) according to *P2RX7* rs3751143 genotype, AA (*n* = 36) vs AC + CC (*n* = 22), in female high-level endurance runners. Higher _T_BMD and _L_BMD but not _LS_BMD in AA than AC + CC genotypes. Error bars denote standard deviation

In the dominant analysis model, female runners with *P2RX7* rs3751143 AA genotype had 4% higher _T_BMD (*P* = 0.052) and 5% higher _L_BMD (*P* = 0.036) than AC + CC genotype female runners but no differences were evident for _LS_BMD (*P* = 0.512) as shown in Fig. 1. Additionally, AA genotype female runners had a higher T-score (1.36 vs 0.81; *P* = 0.047) and Z-score (1.27 vs 0.70; *P* = 0.017) in comparison to AC + CC genotypes.

**Fig. 2:**
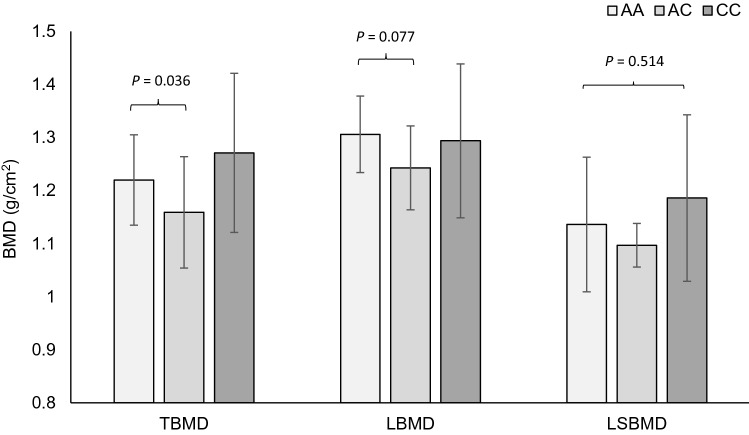
Mean total bone mineral density (_T_BMD), leg bone mineral density (_L_BMD) and lumbar spine bone mineral density (_LS_BMD) according to *P2RX7* rs3751143 genotype, AA (*n* = 36) vs AC (*n* = 19) vs CC (*n* = 3), in female high-level endurance runners. Higher _T_BMD but not _L_BMD in AA than AC genotypes. No differences between genotypes for _LS_BMD. Error bars denote standard deviation

In the additive analysis model, a main effect of *P2RX7* rs3751143 genotype on _T_BMD (*P* = 0.016), _L_BMD (*P* = 0.080), T-score (*P* = 0.018) and Z-score (*P* = 0.013) but not _LS_BMD (*P* = 0.514) existed within the female runners. Following post-hoc analysis, those with AA genotype possessed 5% higher _T_BMD than AC genotypes (*P* = 0.036) but no difference was evident for _L_BMD (*P* = 0.077) as shown in Fig. 2. AA genotypes also had a higher T-score (1.36 vs 0.64; *P* = 0.034) and Z-score (1.26 vs 0.57; *P* = 0.016) than AC genotypes. No genotype-dependent differences for *P2RX7* rs3751143 on any bone phenotype were present within runners in the recessive analysis model (corrected *P* ≥ 0.288).

**Fig. 3:**
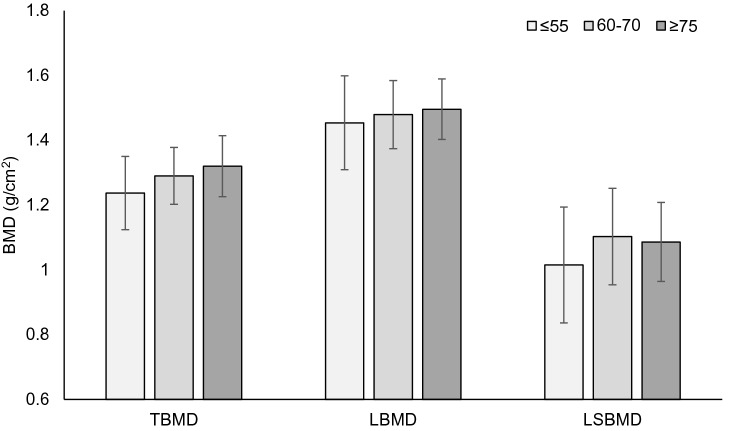
Mean total bone mineral density (_T_BMD), leg bone mineral density (_L_BMD) and lumbar spine bone mineral density (_LS_BMD) according to total genotype score (TGS) group, < 55 (*n* = 7) vs 60–70 (*n* = 32) vs > 75 (*n* = 6), in male high-level endurance runners. No differences between groups defined by TGS for any bone phenotype in male runners. Error bars denote standard deviation

**Fig. 4:**
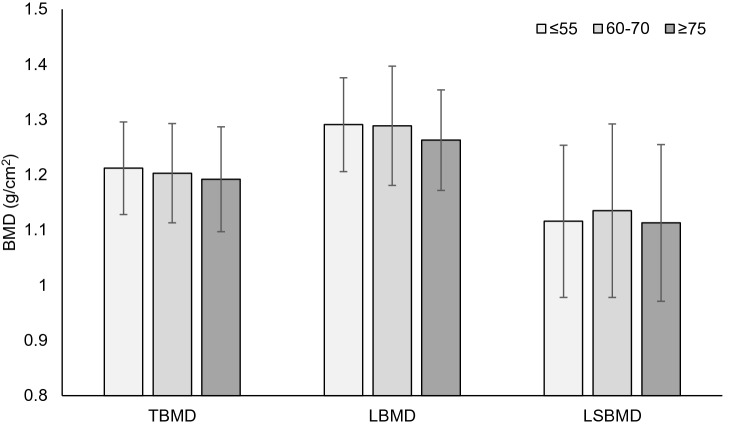
Mean total bone mineral density (_T_BMD), leg bone mineral density (_L_BMD) and lumbar spine bone mineral density (_LS_BMD) according to total genotype score (TGS) group, < 55 (*n* = 13) vs 60–70 (*n* = 34) vs > 75 (*n* = 11), in female high-level endurance runners. No differences between groups defined by TGS for any bone phenotype in female runners. Numbers within bars are number of runners per TGS group and error bars denote standard deviation

No other SNPs, individually or collectively as part of a TGS were associated with any bone phenotypes in men or women after multiple testing correction (corrected *P* ≥ 0.215; Figs. 3 and 4).

Full results from analyses of genotype and bone phenotypes in runners are provided in Tables 1 and 2 in supplementary material.

### Genotype-cohort interactions

**Fig. 5:**
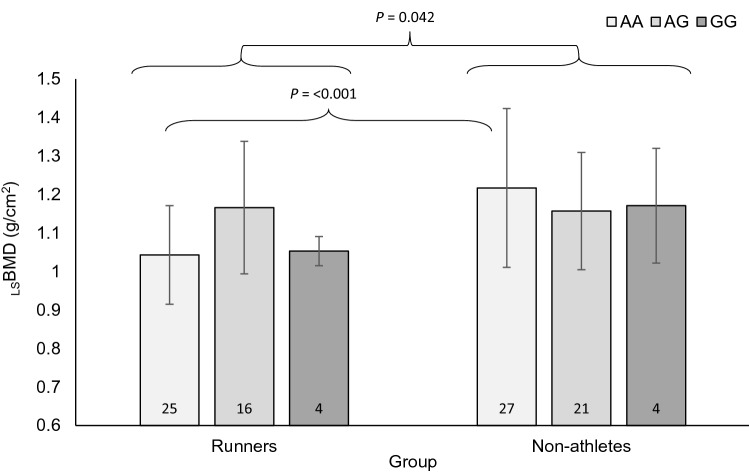
Mean lumbar spine bone mineral density (_LS_BMD) in male runners and non-athletes according to *WNT16* rs3801387 genotype (AA vs AG vs GG). Significant interaction between runner/non-athlete status and genotype (*P* = 0.042), with notably lower _LS_BMD in AA genotype runners than non-athletes (*P* < 0.001). Numbers within bars are number of runners per genotype group and error bars denote standard deviation

A genotype-cohort interaction was evident for *WNT16* rs3801387 and all bone phenotypes in men for both the additive (_T_BMD, *P* = 0.057; _L_BMD, *P* = 0.032; _LS_BMD, *P* = 0.042; T-score, *P* = 0.052 and Z-score, *P* = 0.045; Table 1 in Supplementary Material) and recessive analysis models (_T_BMD, *P* = 0.020; _L_BMD, *P* = 0.009; _LS_BMD, *P* = 0.021; T-score, *P* = 0.040; Z-score, *P* = 0.015). Male runners with *WNT16* rs3801387 AA genotype in comparison to their AA genotype non-athlete counterparts, had lower values for _T_BMD, T-score and Z-score (*P* ≤ 0.014), as well as _LS_BMD, where the largest difference was shown (14%; *P* < 0.001; Fig. 5). In addition, *WNT16* rs3801387 AG + GG genotypes had 5% higher _L_BMD than AG + GG non-athletes (*P* = 0.049). There were no other genotype-cohort interactions in men, and no genotype-cohort interactions in women.

Full genotype-cohort interaction results for the additive model are provided in Tables 1 and 2 in supplementary material.

## Discussion

This study is the first to investigate bone phenotypes and BMD-associated genetic variants in high-level endurance runners and non-athletes. We report novel associations between BMD and *P2RX7* rs3751143 for high-level endurance runners whilst *WNT16* rs3801387 demonstrated genotype-cohort differences in BMD between runners and non-athletes.

In female runners, *P2RX7* rs3751143 AA genotypes had higher _T_BMD, _L_BMD, T-score and Z-score than AC + CC genotypes. Higher BMD in AA genotypes has been reported previously in non-athletes (Wesselius et al. 2013) whilst lower _LS_BMD has been shown in CC homozygote osteoporotic women (Husted et al. 2013). The *P2RX7* rs3751143 C-allele has been associated with osteoclast apoptosis (Ohlendorff et al. 2007) and reduced bone strength as well as stress fracture incidence in elite athletes (Varley et al. 2016), which indicates that the loss-of-function C-allele may reduce BMD. This is the first study to investigate the association of *P2RX7* rs3751143 on these bone phenotypes in an endurance runner cohort, suggesting that possessing a C allele may also negatively impact BMD, similarly to non-athletes. The differences we observed in measured bone phenotypes (except _LS_BMD) between AA and AC + CC genotypes in runners indicates that possessing the AA genotype is beneficial for BMD, particularly at sites where a greater volume of mechanical loading is occurring. Consequently, *P2RX7* rs3751143 may have a greater influence on bone at certain points across the lifespan via interaction with mechanical loading and is thus particularly pertinent when enhancing bone mass in childhood or using exercise to combat BMD loss during ageing. Moreover, some SNPs may have a greater influence on particular bone components. For example, *P2RX7* rs1718119 has been associated with cortical thickness whilst sclerostin (*SOST*) rs1877632 has been associated with trabecular density (Varley et al. 2018). Cortical and trabecular bone adaptation and loss occur at differing rates (Riggs et al. 2008) and thus genotype-dependent differences may influence these phenotypes distinctly.

No significant associations were observed for the *TNFRSF11A* rs3018362 or *TNFRSF11B* rs4355801 SNPs. Both SNPs are reported to influence bone metabolism and BMD within non-athlete populations (Richards et al. 2008; Albagha et al. 2010) but appear unrelated to bone phenotypes within high-level endurance runners. Similarly, *AXIN1* rs9921222, *COMT* rs4680, *LRP5* rs3736228 and *VDR* rs2228570 genotypes did not differ for any bone phenotypes in the current study, but these observations are in contrast with previous literature that has shown potential gene-physical activity/mechanical loading interactions with BMD (Nakamura et al. 2002a; Mitchell et al. 2016). These contrasting findings may be due to inconsistencies in the measurement of physical activity used (e.g. questionnaires), as well as specific genotype-dependent differences in response to various types of mechanical loading. For example, Nakamura et al. (2002a, b) investigated handball, volleyball and jumping athletes, all of whom require movements of high-impact loading and forces that are multi-directional in nature, and are thus very different from the lower impact and cyclical movements completed in endurance running. Consequently, the outcomes of potential gene-mechanical loading interactions may differ.

A genotype-cohort interaction was observed for *WNT16* rs3801387 and all bone phenotypes in men for both the additive and recessive analysis models. Male runners with AA genotype possessed lower _T_BMD, _LS_BMD, T-score and Z-score than AA genotype non-athletes, whilst AG + GG genotype runners had higher _L_BMD than their non-athlete counterparts. Interestingly, AA genotype was most advantageous for BMD in non-athletes but most detrimental for runners, with higher values for all bone phenotypes observed in the AG + GG genotype group. Accordingly, the A allele has been previously associated with lower _LS_BMD, femoral neck BMD and osteoporotic fracture in non-athletes (Estrada et al. 2012). Furthermore, this SNP also interacts with physical activity and _LS_BMD in children (Mitchell et al. 2016). These findings suggest that possessing *WNT16* rs3801387 AA genotype may have a greater impact on BMD in runners than non-athletes, particularly at sites where less loading occurs. Wnt16 is a key regulator of osteoblast-to-osteoclast communication and targeted disruption of Wnt16 in mice results in a 27% loss in bone size and 43–61% loss in bone strength (Zheng et al. 2012). Wnt16 expression is also reported to be influenced by oestrogen receptor signalling in a sex-specific manner with age in mouse tibia (Todd et al. 2015). Oestrogen deficiency has been reported to decrease Wnt16 expression whilst oestrogen replacement increased Wnt16 expression in mouse cortical bone (Alam et al. 2017). Moreover, low oestradiol levels have been reported and associated with reduced BMD in male endurance runners previously (Ackerman et al. 2012). Consequently, it could be hypothesised that interactions between *WNT16* variants (and subsequent Wnt16 expression) and low oestradiol levels in male runners could negatively impact BMD.

No further genotype-cohort interactions for any bone phenotypes were observed in either men or women. The findings for *TNFRSF11A* rs3018362 and *P2RX7* rs3751143 SNPs are therefore in agreement with Varley et al. (2018) who showed no genotype-by-time interactions on bone phenotypes following completion of a 12-week training programme in academy football players.

### Conclusion

This study is the first to investigate bone phenotypes and BMD-associated genetic variants in high-level endurance runners and non-athletes. We report novel associations between *P2RX7* rs3751143 genotype and BMD in female runners, whilst differences in BMD between male runners and non-athletes with the same *WNT16* rs3801387 genotype existed, highlighting a potential genetic interaction with factors common in endurance runners, such as mechanical loading. These findings contribute to our knowledge of genetic associations with BMD and improve our understanding of why some runners have lower BMD than others. Independent replication plus identifying other relevant genetic variations could lead towards more personalised exercising programming, partly based upon genetic information, to manage injury risk and thus improve health and performance in endurance runners.

## Supplementary Information

Below is the link to the electronic supplementary material.

Supplementary file1 (DOCX 25 KB)

Supplementary file2 (DOCX 26 KB)

## Data Availability

The datasets generated during and/or analysed during the current study are available from the corresponding author on reasonable request.

## Abbreviations

ANOVA: Analysis of variance
BMD: Bone mineral density
DXA: Dual-energy X-ray absorptiometry
GWAS: Genome-wide association study
SNP: Single nucleotide polymorphism
TGS: Total genotype score
