# Supplementary material for: Bone mineral density in high-level endurance runners: Part B—genotype-dependent characteristics
Source: Eur J Appl Physiol. 2021 Sep 22;122(1):71–80. doi: 10.1007/s00421-021-04789-z (PMC8748376; doi:10.1007/s00421-021-04789-z)
Supplement: Supplementary file 1 — Supplementary file1 (DOCX 25 KB) [file 421_2021_4789_MOESM1_ESM.docx]

**Supplementary material:**

**Table 1:** Bone phenotype mean (SD) for all 10 SNPs in men with uncorrected *P*-values for the additive model genotype-cohort interaction (INT) and the main effect of genotype within the runners (RUN). N/A and (N/A) indicate that no or only one individual possessed the specific genotype for that SNP.

| **SNP** | **Runners** | | | | | **Non-athletes** | | | | | ***P* - Value** | |
| --- | --- | --- | --- | --- | --- | --- | --- | --- | --- | --- | --- | --- |
|  | **_T_BMD** | **_L_BMD** | **_LS_BMD** | **T-S** | **Z-S** | **_T_BMD** | **_L_BMD** | **_LS_BMD** | **T-S** | **Z-S** | **INT** | **RUN** |
| *AXIN1*  rs9921222  CC  CT  TT | 1.271 (0.061)  1.303 (0.100)  1.249  (0.092) | 1.465 (0.099)  1.492 (0.106)  1.448 (0.124) | 1.039 (0.104)  1.104 (0.152)  1.081 (0.180) | 0.73 (0.57)  1.00 (0.92)  0.50 (0.92) | 0.68 (0.55)  0.98 (0.89)  0.49 (0.89) | 1.335 (0.114)  1.316 (0.127)  1.292 (0.089) | 1.514 (0.124)  1.464 (0.118)  1.462 (0.164) | 1.262 (0.206)  1.182 (0.183)  1.132 (0.134) | 1.28 (1.02)  1.19 (1.20)  0.89 (0.87) | 1.30 (0.92)  1.15 (1.09)  0.92 (0.66) | 0.619  0.459  0.189  0.759  0.602 | 0.272  0.525  0.564  0.283  0.267 |
| *BDNF-AS*  rs6265  CC  CT  TT | 1.289 (0.095)  1.281 (0.098)  1.264 (N/A) | 1.472 (0.108)  1.487 (0.115)  1.468 (N/A) | 1.095 (0.144)  1.077 (0.169)  1.066 (N/A) | 0.87 (0.88)  0.80 (0.93)  0.70 (N/A) | 0.84 (0.84)  0.79 (0.92)  0.60 (N/A) | 1.319 (0.901)  1.301 (0.156)  1.400 (N/A) | 1.494 (0.110)  1.434 (0.164)  1.596 (N/A) | 1.203 (0.168)  1.156 (0.211)  1.300 (N/A) | 1.22 (0.88)  0.94 (1.40)  1.90 (N/A) | 1.16 (0.76)  1.01 (1.27)  2.00 (N/A) | 0.757  0.278  0.792  0.718  0.669 | 0.946  0.904  0.926  0.956  0.957 |
| *COL1A1*  rs1800012  CC  CA  AA | 1.301 (0.085)  1.259 (0.109)  1.226 (N/A) | 1.501 (0.100)  1.434 (0.117)  1.438 (N/A) | 1.092 (0.139)  1.076 (0.180)  1.151 (N/A) | 0.99 (0.79)  0.59 (1.03)  0.30 (N/A) | 0.97 (0.77)  0.57 (0.97)  0.20 (N/A) | 1.328 (0.107)  1.255 (0.133)  1.360 (0.093) | 1.496 (0.118)  1.406 (0.165)  1.485 (0.126) | 1.200 (0.186)  1.106 (0.137)  1.360 (0.149) | 1.29 (0.97)  0.50 (1.27)  1.53 (0.81) | 1.24 (0.89)  0.65 (1.11)  1.50 (0.70) | 0.515  0.837  0.494  0.455  0.535 | 0.323  0.143  0.870  0.293  0.261 |
| *COMT*  rs4680  GG  GA  AA | 1.300 (0.093)  1.304 (0.091)  1.257 (0.096) | 1.516 (0.111)  1.489 (0.104)  1.444 (0.108) | 1.080 (0.119)  1.087 (0.116)  1.092 (0.200) | 0.98 (0.87)  1.02 (0.82)  0.58 (0.93) | 0.96 (0.84)  0.99 (0.79)  0.55 (0.89) | 1.294 (0.101)  1.318 (0.101)  1.323 (0.150) | 1.461 (0.130)  1.488 (0.108)  1.463 (0.178) | 1.127 (0.223)  1.201 (0.161)  1.211 (0.193) | 0.90 (0.98)  1.23 (0.96)  1.13 (1.36) | 0.97 (0.85)  1.15 (0.86)  1.20 (1.21) | 0.432  0.581  0.735  0.542  0.393 | 0.286  0.225  0.981  0.292  0.258 |
| *LRP5*  rs3766228  CC  CT  TT | 1.294 (0.097)  1.274 (0.093)  1.245 (N/A) | 1.480 (0.111)  1.478 (0.109)  1.397 (N/A) | 1.094 (0.157)  1.087 (0.144)  0.940 (N/A) | 0.91 (0.88)  0.74 (0.91)  0.50 (N/A) | 0.88 (0.85)  0.72 (0.88)  0.50 (N/A) | 1.330 (0.114)  1.281 (0.110)  N/A | 1.491 (0.142)  1.442 (0.101)  N/A | 1.211 (0.199)  1.141 (0.128)  N/A | 1.31 (1.06)  0.77 (1.04)  N/A | 1.27 (0.93)  0.82 (0.96)  N/A | 0.521  0.366  0.391  0.388  0.470 | 0.736  0.760  0.615  0.778  0.779 |
| *P2RX7*  rs3751143  AA  AC  CC | 1.289 (0.078)  1.267 (0.128)  1.408 (N/A) | 1.479 (0.104)  1.462 (0.122)  1.621 (N/A) | 1.092 (0.143)  1.076 (0.181)  1.100 (N/A) | 0.88 (0.72)  0.64 (1.21)  2.00 (N/A) | 0.86 (0.71)  0.61 (1.14)  1.90 (N/A) | 1.299 (0.103)  1.342 (0.140)  1.389 (0.048) | 1.458 (0.125)  1.501 (0.147)  1.602 (0.044) | 1.174 (0.189)  1.199 (0.163)  1.383 (0.086) | 1.03 (0.99)  1.31 (1.27)  1.80 (0.42) | 1.01 (0.87)  1.30 (1.15)  1.85 (0.21) | 0.390  0.556  0.583  0.473  0.411 | 0.342  0.376  0.949  0.305  0.302 |
| *TNFRSF11A*  rs3018362  GG  GA  AA | 1.297 (0.107)  1.272 (0.076)1.249 (0.027) | 1.477 (0.128)  1.480 (0.077)  1.464 (0.041) | 1.110 (0.166)  1.063 (0.126)  1.013 (0.102) | 0.94 (0.99)  0.73 (0.74)  0.53 (0.25) | 0.90 (0.93)  0.73 (0.77)  0.47 (0.21) | 1.358 (0.130)  1.270 (0.092)  1.336 (0.074) | 1.532 (0.130)  1.415 (0.121)  1.519 (0.072) | 1.222 (0.195)  1.156 (0.126)  1.204 (0.102) | 1.48 (1.15)  0.80 (1.00)  1.30 (0.67) | 1.45 (0.94)  0.78 (0.77)  1.37 (0.21) | 0.286  0.057  0.753  0.452  0.312 | 0.575  0.973  0.436  0.644  0.628 |
| *TNFRSF11B*  rs4355801  AA  AG  GG | 1.294 (0.081)  1.276 (0.107)  1.294 (0.086) | 1.487 (0.116)  1.471 (0.114)  1.479 (0.123) | 1.065 (0.097)  1.096 (0.194)  1.102 (0.094) | 0.94 (0.75)  0.75 (1.00)  0.92 (0.80) | 0.89 (0.78)  0.73 (0.95)  0.92 (0.76) | 1.315 (0.111)  1.310 (0.115)  1.320 (0.123) | 1.473 (0.110)  1.487 (0.135)  1.466 (0.151) | 1.199 (0.183)  1.162 (0.185)  1.213 (0.182) | 1.28 (1.08)  1.05 (1.04)  1.13 (1.15) | 1.15 (0.89)  1.08 (1.00)  1.17 (0.99) | 0.966  0.844  0.688  0.970  0.968 | 0.817  0.911  0.792  0.796  0.795 |
| *VDR*  rs2228570  GG  GA  AA | 1.296 (0.088)  1.269 (0.104)  1.299 (0.090) | 1.477 (0.097)  1.462 (0.123)  1.512 (0.104) | 1.086 (0.136)  1.070 (0.140)  1.131 (0.210) | 0.94 (0.79)  0.68 (1.00)  0.99 (0.84) | 0.91 (0.77)  0.66 (0.95)  0.96 (0.81) | 1.304 (0.115)  1.319 (0.110)  1.328 (0.148) | 1.462 (0.148)  1.483 (0.120)  1.494 (0.144) | 1.202 (0.190)  1.177 (0.180)  1.204 (0.191) | 1.00 (1.08)  1.23 (1.05)  1.20 (1.29) | 1.01 (0.94)  1.20 (0.94)  1.17 (1.19) | 0.682  0.771  0.923  0.544  0.538 | 0.632  0.573  0.652  0.597  0.589 |
| *WNT16*  rs3801387  AA  AG  GG | 1.266 (0.088)  1.320 (0.103)  1.270 (0.066) | 1.448 (0.107)  1.519 (0.106)  1.492 (0.084) | 1.043 (0.128)  1.166 (0.172)  1.053 (0.037) | 0.65 (0.84)  1.17 (0.94)  0.73 (0.61) | 0.62 (0.81)  1.14 (0.89)  0.70 (0.62) | 1.342 (0.123)  1.287 (0.101)  1.277 (0.979) | 1.507 (0.122)  1.443 (0.136)  1.442 (0.161) | 1.217 (0.206)  1.157 (0.152)  1.171 (0.149) | 1.33 (1.09)  0.84 (0.97)  1.43 (1.35) | 1.35 (1.00)  0.90 (0.87)  0.80 (0.81) | 0.057  **0.032***  **0.042***  0.052  **0.045*** | 0.198  0.113  **0.030***  0.180  0.154 |
