# Supplementary material for: Bone mineral density in high-level endurance runners: Part B—genotype-dependent characteristics
Source: Eur J Appl Physiol. 2021 Sep 22;122(1):71–80. doi: 10.1007/s00421-021-04789-z (PMC8748376; doi:10.1007/s00421-021-04789-z)
Supplement: Supplementary file 2 — Supplementary file2 (DOCX 26 KB) [file 421_2021_4789_MOESM2_ESM.docx]

**Table 2:** Bone phenotype mean (SD) for all 10 SNPs in women with uncorrected *P*-values for the additive model genotype-cohort interaction (INT) and the main effect of genotype within the runners (RUN). N/A and (N/A) indicate that no or only one individual possessed the specific genotype for that SNP.

| **SNP** | **Runners** | | | | | **Non-athletes** | | | | | ***P* - Value** | |
| --- | --- | --- | --- | --- | --- | --- | --- | --- | --- | --- | --- | --- |
|  | **_T_BMD** | **_L_BMD** | **_LS_BMD** | **T-S** | **Z-S** | **_T_BMD** | **_L_BMD** | **_LS_BMD** | **T-S** | **Z-S** | **INT** | **RUN** |
| *AXIN1*  rs9921222  CC  CT  TT | 1.200 (0.098)  1.196 (0.718)  1.223 (0.091) | 1.286 (0.103)  1.290 (0.100)  1.273 (0.095) | 1.147 (0.174)  1.093 (0.123)  1.132 (0.117) | 1.10 (1.15)  1.06 (0.87)  1.40 (1.09) | 1.05 (1.03)  0.99 (0.66)  1.15 (0.96) | 1.216 (0.114)  1.177 (0.107)  1.192 (0.103) | 1.249 (0.138)  1.218 (0.110)  1.262 (0.127) | 1.169 (0.146)  1.186 (0.199)  1.153 (0.176) | 1.28 (1.33)  0.82 (1.28)  1.00 (1.20) | 1.40 (1.17)  0.79 (1.12)  1.05 (1.12) | 0.586  0.549  0.529  0.568  0.423 | 0.690  0.901  0.472  0.666  0.898 |
| *BDNF-AS*  rs6265  CC  CT  TT | 1.193 (0.095)  1.218 (0.082)  1.227 (0.035) | 1.276 (0.101)  1.305 (0.103)  1.282 (0.057) | 1.114 (0.140)  1.153 (0.165)  1.135 (0.183) | 1.02 (1.11)  1.33 (0.98)  1.45 (0.42) | 0.97 (0.99)  1.18 (0.77)  1.30 (0.29) | 1.206 (0.113)  1.166 (0.097)  1.158 (0.137) | 1.253 (0.128)  1.204 (0.107)  1.185 (0.042) | 1.210 (0.180)  1.124 (0.160)  1.031 (0.205) | 1.16 (1.33)  0.71 (1.15)  0.60 (1.70) | 1.19 (1.16)  0.73 (1.08)  0.60 (1.70) | 0.219  0.189  0.091  0.217  0.199 | 0.555  0.600  0.666  0.514  0.616 |
| *COL1A1*  rs1800012  CC  CA  AA | 1.191 (0.092)  1.240 (0.066)  N/A | 1.282 (0.102)  1.292 (0.092)  N/A | 1.117 (0.154)  1.158 (0.130)  N/A | 1.00 (1.09)  1.60 (0.75)  N/A | 0.95 (0.94)  1.37 (0.66)  N/A | 1.198 (0.110)  1.177 (0.106)  N/A | 1.239 (0.121)  1.226 (0.124)  N/A | 1.174 (0.170)  1.178 (0.198)  N/A | 1.07 (1.30)  0.84 (1.24)  N/A | 1.08 (1.18)  0.89 (1.10)  N/A | 0.094  0.620  0.583  0.083  0.154 | 0.074  0.757  0.371  0.080  0.128 |
| COMT  rs4680  GG  GA  AA | 1.218 (0.101)  1.196 (0.093)  1.198 (0.069) | 1.289 (0.108)  1.281 (0.104)  1.285 (0.089) | 1.135 (0.183)  1.130 (0.155)  1.115 (0.109) | 1.33 (1.20)  1.06 (1.12)  1.09 (0.82) | 1.12 (1.00)  1.03 (0.98)  1.03 (0.72) | 1.214 (0.122)  1.178 (0.106)  1.192 (0.102) | 1.251 (0.136)  1.220 (0.111)  1.246 (0.128) | 1.211 (0.145)  1.168 (0.184)  1.556 (0.201) | 1.27 (1.45)  0.84 (1.24)  1.03 (1.19) | 1.33 (1.23)  0.88 (1.13)  0.98 (1.11) | 0.936  0.871  0.867  0.935  0.745 | 0.714  0.970  0.926  0.704  0.945 |
| *LRP5*  rs3766228  CC  CT  TT | 1.217 (0.089)  1.167 (0.079)  1.235 (0.091) | 1.300 (0.092)  1.240 (0.105)  1.359 (0.066) | 1.148 (0.147)  1.087 (0.152)  1.047 (0.112) | 1.31 (1.05)  0.72 (0.95)  1.55 (1.06) | 1.21 (0.87)  0.67 (0.89)  1.30 (0.71) | 1.196 (0.111)  1.178 (0.105)  1.155 (N/A) | 1.230 (0.121)  1.253 (0.124)  1.149 (N/A) | 1.182 (0.173)  1.161 (0.202)  1.123 (N/A) | 1.05 (1.31)  0.84 (1.25)  0.60 (N/A) | 1.06 (1.17)  0.94 (1.15)  0.50 (N/A) | 0.634  0.097  0.836  0.629  0.510 | 0.124  0.061  0.277  0.129  0.109 |
| *P2RX7*  rs3751143  AA  AC  CC | 1.220 (0.085)  1.159 (0.072)  1.271 (0.127) | 1.306 (0.106)  1.243 (0.080)  1.294 (0.042) | 1.137 (0.151)  1.098 (0.145)  1.187 (0.157) | 1.36 (1.00)  0.62 (0.88)  1.90 (1.47) | 1.27 (0.82)  0.57 (0.82)  1.50 (1.30) | 1.207 (0.106)  1.164 (0.116)  1.129 (0.379) | 1.251 (0.119)  1.208 (0.126)  1.163 (0.088) | 1.200 (0.171)  1.146 (0.187)  0.999 (0.154) | 1.19 (1.25)  0.65  (1.37)  0.30 (0.46) | 1.78 (1.15)  0.72 (1.18)  0.53 (0.55) | 0.232  0.601  0.194  0.270  0.447 | **0.016***  0.080  0.514  **0.018***  **0.013*** |
| *TNFRSF11A*  rs3018362  GG  GA  AA | 1.216 (0.076)  1.200 (0.101)  1.158 (0.095) | 1.296 (0.094)  1.278 (0.112)  1.256 (0.088) | 1.128 (0.137)  1.152 (0.178)  1.063 (0.106) | 1.30 (0.91)  1.11 (1.19)  0.60 (1.14) | 1.22 (0.67)  0.96 (1.09)  0.63 (1.09) | 1.180 (0.123)  1.197 (0.090)  1.213 (0.161) | 1.224 (0.130)  1.237 (0.109)  1.286 (0.170) | 1.165 (0.194)  1.188 (0.172)  1.143 (0.153) | 0.86 (1.46)  1.07 (1.05)  1.23 (1.90) | 0.93 (1.24)  1.06 (1.00)  1.18 (1.90) | 0.350  0.374  0.921  0.348  0.389 | 0.244  0.565  0.374  0.237  0.209 |
| *TNFRSF11B*  rs4355801  AA  AG  GG | 1.201 (0.083)  1.209 (0.084)  1.191 (0.110) | 1.303 (0.110)  1.289 (0.096)  1.247 (0.122) | 1.131 (0.155)  1.123 (0.153)  1.128 (0.140) | 1.13 (0.97)  1.22 (1.00)  0.98 (1.30) | 1.01 (0.92)  1.14 (0.82)  0.93 (1.08) | 1.238 (0.093)  1.170 (0.096)  1.196 (0.132) | 1.295 (0.108)  1.220 (0.109)  1.219 (0.142) | 1.187 (0.144)  1.144 (0.171)  1.224 (0.209) | 1.56 (1.07)  0.75 (1.14)  1.04 (1.56) | 1.65 (0.92)  0.75 (1.06)  1.05 (1.31) | 0.213  0.432  0.616  0.215  0.087 | 0.836  0.307  0.982  0.797  0.776 |
| *VDR*  rs2228570  GG  GA  AA | 1.196 (0.074)  1.210 (0.098)  1.201 (0.101) | 1.276 (0.090)  1.299 (0.105)  1.271 (0.109) | 1.107 (0.107)  1.147 (0.183)  1.125 (0.152) | 1.07 (0.88)  1.23 (1.16)  1.11 (1.21) | 1.01 (0.81)  1.07 (0.97)  1.12 (0.98) | 1.167 (0.092)  1.219 (0.114)  1.150 (0.106) | 1.220 (0.108)  1.249 (0.133)  1.218 (0.109) | 1.139 (0.174)  1.211 (0.171)  1.134 (0.204) | 0.71 (1.09)  1.33 (1.32)  0.49 (1.28) | 0.83 (1.04)  1.30 (1.17)  0.49 (1.12) | 0.432  0.990  0.790  0.418  0.245 | 0.862  0.649  0.651  0.870  0.948 |
| *WNT16*  rs3801387  AA  AG  GG | 1.211 (0.086)  1.184 (0.101)  1.204 (0.059) | 1.299 (0.091)  1.245 (0.120)  1.304 (0.066) | 1.147 (0.136)  1.101 (0.185)  1.071 (0.109) | 1.24 (1.03)  0.92 (1.21)  1.18 (0.71) | 1.14 (0.90)  0.84 (1.00)  1.13 (0.54) | 1.184 (0.102)  1.195 (0.116)  1.195 (0.110) | 1.234 (0.115)  1.229 (0.130)  1.255 (0.117) | 1.183 (0.216)  1.179 (0.157)  1.145 (0.158) | 0.92 (1.20)  1.04 (1.36)  1.01 (1.29) | 0.86 (1.03)  1.08 (1.24)  1.19 (1.20) | 0.658  0.555  0.814  0.670  0.472 | 0.603  0.172  0.370  0.570  0.534 |
